# “Lay epidemiology”: an important factor in Danish parents’ decision of whether to allow their child to receive a BCG vaccination. A qualitative exploration of parental perspective

**DOI:** 10.1186/s12887-017-0944-3

**Published:** 2017-11-21

**Authors:** Gitte Thybo Pihl, Helle Johannessen, Jette Ammentorp, Jane Schmidt Jensen, Poul-Erik Kofoed

**Affiliations:** 1 Department of Paediatrics, Lillebaelt Hospital, Skovvangen 2-8, DK-6000 Kolding, Denmark; 2 Faculty of Health Sciences, University of Southern Denmark, J.B. Winsløws Vej 19, 3. sal, DK-5000 Odense C, Denmark; 3 Health Services Research Unit, Lillebaelt Hospital, Kabbeltoft 25, DK-7100 Vejle, Denmark

**Keywords:** Decision making, Lay epidemiology, Risk evaluation, Patient-provider communication, Vaccine safety concerns, Values, Heterologous immunity.

## Abstract

**Background:**

Vaccination is used worldwide to prevent infectious diseases. However, vaccination programmes in western countries face challenges in sustaining high coverage rates. The aim of this study was to explore how parents in Denmark make a decision about whether to allow their child to receive a Bacille Calmette Guerin vaccine at birth for the purpose of achieving non-specific effects on the immune system.

**Methods:**

A total of five focus groups were conducted with expectant mothers and fathers. Written information about the vaccine and information about the hypothesis of non-specific effects of the vaccine were delivered in order to discuss considerations and determinants of parents’ decisions.

**Results:**

Heritable factors and the possibility of stimulating the immune system of the child to achieve less atopic diseases and fewer infections were identified as arguments in favour of receiving the BCG vaccine. Arguments against receiving BCG mainly focused on concerns about its described and non-described side effects. Both arguments for and arguments against the vaccine were seen as parents attempt to make an individual risk evaluation for their child. Attitudes and beliefs in the local network were identified as important for parents’ decisions.

**Discussion:**

It is discussed how “lay epidemiology” characterizes parents’ risk evaluation as an individual addition to the population-based risk declaration. It is furthermore discussed how health professionals should engage with both the empirical element and the value element of “Lay epidemiology”.

**Conclusion:**

“Lay epidemiology” forms the basis for the parental decision of whether to allow their child to receive a BCG vaccination. Attitudes and beliefs about the causes and distribution of illnesses in the family or local network influence parents’ risk evaluations. It would be ideal for parents if health professionals focused their communication about the BCG vaccine on individual risk evaluations.

## Background

Vaccination is used worldwide to prevent infectious diseases, and the establishment of vaccination programmes throughout the world is a major public health achievement [1]. However, vaccination programmes in western countries face challenges in sustaining high coverage rates [http://www.who.int/immunization/research/implementation/en/]. The most common barrier to paediatric immunization is parental concerns about the negative side effects of vaccines [2–4]. In addition, some parents believe that the immune system becomes stronger by being challenged by the natural infectious disease, that the immune system might be overwhelmed if exposed to too many vaccines, or that additives in the vaccines might be harmful [5]. Accordingly, paediatricians are concerned if these barriers to vaccination are based on an insufficient level of knowledge and how to address and overcome these barriers [6]. Research that has explored parental knowledge, attitudes, and beliefs about vaccination suggests that health care providers must support each individual parent in making decisions about having their children immunized [7]. Therefore, it is important to study parents’ considerations about vaccines in order to learn how to support parental decision making.

The Bacille Calmette Guerin (BCG) vaccine has been used for almost 100 years to prevent tuberculosis [8] and is part of childhood immunization programmes in many countries, but it was removed from the Danish vaccination programme 30 years ago due to the low prevalence of tuberculosis in Denmark. However, epidemiological studies in Africa have found lower mortality and morbidity and less atopic diseases among children immunized with BCG [9–12]. Whether these positive non-specific effects of BCG also occur in western societies is being tested by a large prospective randomized clinical trial in Denmark, the Danish Calmette Study [http://calmette-studiet.dk/Om%20Studiet/Studiemateriale/Protokol.aspx]. The Danish Calmette Study is a multicentre study with cooperation between Rigshospitalet in Copenhagen, Hvidovre and Kolding Hospitals. The study was designed as a clinical trial with telephone interviews, clinical investigations and register-based follow-up [http://calmette-studiet.dk/Om%20Studiet/Studiemateriale/Protokol.aspx]. Even if this trial gives convincing evidence of positive non-specific effects of BCG immunization, it might be difficult to implement the BCG vaccine if parents are reluctant. Therefore, before initiating the clinical trial, we investigated how parents may make the decision of whether to allow their child to receive a BCG vaccination at birth, which is given to achieve possible non-specific effects on the immune system.

## Methods

### Participants and research setting

Before enrollment in the clinical trial began, five focus groups were conducted with expectant mothers and fathers for the purpose of discussing the considerations for and against letting their new-born child be vaccinated with BCG. Parents participating in antenatal classes at Kolding Hospital during the summer of 2012 were invited to the focus groups by one of the co-authors of this paper (JSJ), who also facilitated the groups. Expectant mothers were invited to participate in antenatal courses in the last half of their pregnancy. Most first-time mothers and some second-time mothers took the courses. Fathers were only invited to participate in a few of the sessions.

In addition to the facilitator of the focus groups, one observer was present. All sessions were audio-recorded with written consent from the participants. The observer took notes during the session to identify important themes and arguments. Four focus groups were conducted with three, eight, four and three expectant mothers, respectively, and one focus group was conducted with four expectant fathers. The reason for having separate focus groups was to allow for different considerations of expectant mothers and expectant fathers to be uncovered. Four of the participants were second-time mothers. One focus group with expectant first-time fathers was conducted. The mean age of participants was 28.9 years, with a minimum age of 23 years and a maximum age of 37 years. The socio-economic background of the participants ranged from no education to a college education and from no work to working as a managing director.

**Fig. 1:**
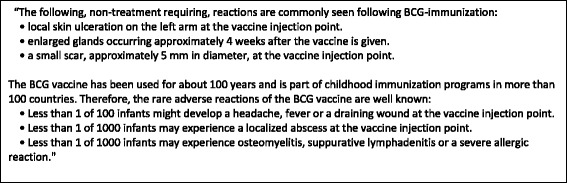
Description of the adverse reactions presented to the participants in the focus groups

**Fig. 2:**
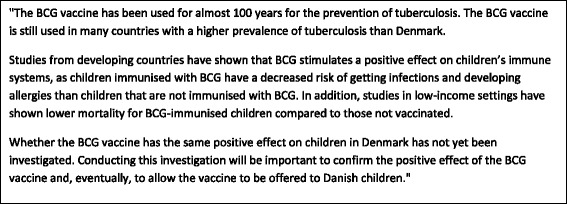
Background information presented to the participants in the focus groups

Before the focus group started, the facilitator provided written information on the BCG vaccine (Fig. 1) and the aim of the Calmette Study (Fig. 2). The parents had time to read the information and were then asked about their considerations regarding whether to allow their new-born child to be immunized with BCG and what would determine their final decision.

The sessions were audio-recorded and transcribed word-for-word. As described by Giorgi and Malterud [13], the analysis process was started by gaining an overall impression of the data from all focus groups and then identifying meaning units. After writing and reading all meaning units, the units were categorized into three groups: “arguments in favour of receiving the BCG vaccine”, “arguments against receiving the BCG vaccine” and “decisional conflicts”. The concept “lay epidemiology” was used in the discussion to understand parental risk evaluation.

## Results

The results were categorized under three main themes, with subheadings structuring the arguments under each main theme.

### Arguments in favour of receiving the BCG vaccine

Statements in favour of receiving the BCG vaccine were phrased very similar to that of the written information about the Danish Calmette Study. When focusing on the beneficial effects of the vaccine, the negative side effects were considered to have minor importance. Other favourable arguments focused on the fact that the vaccine had been used for almost 100 years without causing serious adverse events.

#### Stimulating the immune system

One group of arguments focused on the possible beneficial effects of the BCG vaccine on the immune system, which the participants related to concrete experiences with illness and disease in their own family:

*“There has really been a lot of disease in my part of our family, so I think an augmented immune system would be beneficial in my case.”.*

*“What captured my interest was this idea about the immune system because I have had sinusitis twice a year. I get sick from almost anything, so this [vaccine] would definitely be interesting.”.*

*“... I'm a second-time mother, and when our first child started day care, he was sick all the time, so if you could do anything about that, I mean like with this vaccine, then I would definitely think it could make a difference, also in relation to quality of life when they are starting in day care.”.*

These reflections focused on experiences with disease in the participants own families, including an improved quality of life when children are less sick, and problems regarding taking care of sick children when working. Parents predicted the potency of the immune system of their future child on the basis of experiences with sickness in their own family.

#### Atopic disease

Similarly, parents predicted the risk of their future child to suffer from asthma or eczema on the basis of experiences in their own family.

*“Now I know that my husband’s allergies and asthma are heritable ... if it [the vaccine] can diminish the effect on these heritable factors, I think it would be important.”.*

*“No, I would not hesitate because I have both asthma, allergies and eczema. So I would, if I could, I would do everything possible for the child to avoid that. That is for certain.”.*

The parents knew that allergies and asthma are heritable. They argued that if the vaccine could decrease the risk for their child to contract these disorders, despite their hereditary nature, the vaccine would be beneficial.

#### ‘Risk of negative side effects is negligible compared to the possible benefits’

When speaking with parents about negative side effects of the BCG vaccine, some considered the risk very low compared to the possible benefits of the BCG vaccine.

*“If there had been something other than these minor wounds and then one out of 1000 getting an abscess or so, that's nothing. If there had been something much worse, if you could become hemiplegic after receiving it or something crazy like that, then I think I would say no. But there isn't. It is small events, and it is treatable.”.*

#### An old, well-established vaccine

Many statements emphasized the fact that the BCG vaccine has been used for almost a century without causing serious adverse reactions.

*“I think it’s a good point, too, that it has been used for so long without any incidences; nothing serious appeared. That’s a heavy argument.”*

### Arguments against receiving the BCG vaccine

Parental arguments against BCG immunization mainly focused on its negative side effects; however, the scar caused by the vaccination and the fact that it might be healthier or more safe to avoid being vaccinated were also mentioned.

#### Concerns about side effects

Some of the participants were concerned about non-described negative side effects, and this accounted for a major part of the discussions in the focus groups. Below are a few examples:

*“If it had side effects that could not be readily treated or that could cause serious injury, I would strongly consider saying no.”*

*“I will not risk that my baby gets Down's syndrome from a vaccine or gets some serious injury to the nervous system or something like that.”.*

Some parents feared that severe negative side effects that health professionals had not considered may occur. Furthermore, the parents expressed doubts about the written information provided and asked whether there could be more side effects than those described:

*“What really makes me uncomfortable is … aren’t there any more side effects than those written here? Is it then because they don’t know if there are more side effects or is it because they just want people to get the vaccine? Well, you hear so much about how you trust people and then are getting cheated in these TV programs.”*

Finally, a few parents assessed the risk of the negative side effects of the vaccine differently when the vaccination was being used to achieve non-specific positive side effects rather than to protect against a severe disease.

#### The scar

After BCG vaccination, most children develop a small scar on their upper left arm at the vaccine injection site. Some parents mention this scar as an argument against vaccinating their children.

*“The only thing that could keep me from doing this is the wound and the scar afterwards. What will it look like?”*

A few parents argued that a cosmetic problem is secondary to the beneficial effect of the vaccine on the immune system, but for others, the scar was a reason for not allowing their children to be vaccinated.

#### It is more natural to avoid it

A few parents believe it is more natural to avoid vaccination; they implicitly understand avoiding vaccination as healthier or safer.

*“I think it's more natural to avoid the vaccine, and then I just don't want to put that much into the body - medicine and that kind.”.*

### Decisional conflicts

Two main themes appeared in the meaning units group “decisional conflicts”: attitudes and beliefs in the parents’ network and the need for support from a health professional to make their decision.

#### Attitudes and beliefs in the network

Several parents stated that persons in their network having had vaccination experiences with negative side effects and the attitudes of persons in their network would greatly influence their decision about whether to allow their child to be immunized.

*“If someone had bad experiences with this vaccine, of course it would affect me, especially if they were close to me.”.*

*“I can understand that people refuse if they have a close experience with these vaccines causing brain injury or death, but what is the risk for oneself? I don't know.”.*

It is remarkable, that the closeness of the persons that had negative experiences to the parents has an impact on the parents’ decision. Additionally, it seems that the African research described in the written information from the Danish Calmette Study was considered less important.

*“Research from developing countries, that's kind of far away. We need something closer.”.*

#### Help from a health professional to make a decision

When asked what would be crucial for making a decision, several parents stated that they needed to talk with a health-professional.

*“From what is written here I don't think the side effects are something to worry about, but I would definitely ask more about it before I could make a decision. And then I would ask about experiences.”.*

A father in one of the focus groups read the written information about the negative side effects of the BCG vaccine and replied as follows:

*“What is meant by ‘one in 1000’? I don’t know if my child will be the one or one of the 999.”.*

## Discussion and conclusion

### Discussion

#### “Lay epidemiology” in parental decision making

When analysing the data from the focus-groups, we realized that many parents made a risk evaluation based on the occurrence of sickness in their family and their network to determine if their child could benefit from BCG-immunization. The parents used cases from their network and their family to develop a hunch of how healthy or sick their expectant child would be. The cases were used either as an argument for the vaccine or as an argument against the vaccine.

By searching the literature for an understanding of this phenomenon, the theory of “lay epidemiology” was found to be quite helpful. The term “lay epidemiology” is a theory of risk evaluation and shows how people perform a risk evaluation based on experiences in their network. “Lay epidemiology” is defined by Davison et al. as: “*a scheme in which individuals interpret health risks through the routine observation and discussion of cases of illness and death in personal networks and in the public arena, as well as from formal and informal evidence arising from other sources, such as television and magazines*” [14]. Davison et al. showed how persons in a local community in Wales interpret an individual’s risk of developing a heart disease by observing and discussing the causes for contracting a heart disease in their personal network and by evaluating cases described in the press [14]. The closer a person’s relationship is to a case and the greater the case’s similarity to their own situation, the more weight they give to that case in their own risk evaluation. The parents in the focus groups made similar risk evaluations based on sickness and adverse reactions to vaccines in their network and family.

#### “Lay epidemiology” as an individual addition to population-based risk evaluation

How health professionals should engage with “lay epidemiology” has been widely discussed [15–18]. Several authors have discussed “lay epidemiology” as a barrier to public health messages, arguing that lay epidemiology causes people to disregard health messages. In contrast, Rose et al. discussed the fact that public health messages are based on knowledge of populations but do not tell an individual exactly how much he/she could benefit from a change in lifestyle: *“In mass prevention each individual has usually only a small expectation of benefit, and this small benefit can easily be outweighed by a small risk”* [19]. It is well understood in “lay epidemiology” that reducing the incidence of a disease in a population is not the same as reducing the risk for each individual person. “Lay epidemiology” can be seen as an attempt to make a risk evaluation at the individual level, whereas scientific epidemiology attempts to make a risk evaluation at the population level. This is described very precisely in the following statement: *“What is meant by ‘one in 1000’? I don’t know if my child will be the one or one of the 999.”* In terms of “lay epidemiology”, this statement could be seen as a father doubting the information provided by the declaration of incidence about the risk of his baby experiencing a negative side effect. Thus, he asks for a more individual risk assessment.

One of the parents stated that he would ask the health professional about his “experiences”. This could be seen as a wish to make statistical information more concrete and interpretable at the level of the individual. “Experiences” describe individuals in real-life-situations, whereas statistical information describes numbers and cohorts. Hunt and Emslie expanded on the discussion of “lay epidemiology” vs. scientific epidemiology. They argue that context and complexity are considered important in “lay epidemiology”, while scientific epidemiology attempts to simplify factors; this is described in the following quote, *“However, observations of the links between a lifetime of experiences and subsequent health events (including mortality) within the family offer the lay epidemiologist potential for more complex theorizing based on extensive and detailed knowledge about factors or experiences which could increase risk, or be potential confounders”* [18]. Therefore, “lay epidemiology” should not be seen as a barrier for public health messages, but rather as an important addition to knowledge. Parents assessing whether their child can benefit from the BCG vaccine based on their experiences with diseases in their own family can be seen as making a complex judgement based on detailed knowledge of heritable factors in their family. Conversely, some concerns about negative side effects could be seen as completely unfounded, and the question of how health professionals should deal with this issue remains unclear.

#### Two importantly different elements in “lay epidemiology”

Allmark and Todd described how health professionals can increase the effectiveness of public health messages by focusing on two important elements of “lay epidemiology”: the empirical element and the value element [20].

The empirical element consists of lay beliefs about causes of illness and management of risk [20]. It recognises that public health messages are sometimes exaggerated or even false, since prevention on a population level is not the same as prevention at the individual level [19]. However, the empirical element in lay epidemiology is based on incomplete beliefs and might sometimes result in false conclusions [20]. Hence, scientific epidemiology is necessary to help prevent such false conclusions. An obvious example of a false conclusion is a parent’s fear of their child developing Down’s syndrome as a negative side effect of a vaccine. To correct parents’ concerns about negative side effects, it is important to discuss their beliefs and to recognize them as important to the parents. Because some parents question whether additional negative side effects other than those described by health professionals may occur, it is important to avoid claiming that scientific knowledge is the only and whole truth. Instead, health professionals should explain to parents that the scientifically based information about negative side effects is the best knowledge we have at present and should be willing to discuss the limitations of this knowledge. Health professionals can correct the conclusion that a baby can get Down’s syndrome from a vaccine, but concerns about long-term side effects of vaccines are more difficult to reject due to the lack of evidence of long-term side effects. Health professionals can explain the scientific rationale behind vaccination and then allow the parents to evaluate the information for themselves.

The value element of “lay epidemiology” consists of “*values about the place of health and risks to health in a good life*” [20]. It is also considered an “all-things-considered” view, where personal values and the person’s entire life-situation are considered. The aversion toward putting drugs and vaccines into the body that some parents express is related to their values, as is the consideration of the scar that develops after BCG vaccination as a major or minor side effect. Values are personal and require respect. Respect is necessary to conduct a dialogue about personal values.

Thus, is it important to recognize both the respectful dialogue about the value element and the need for correction to the empirical element of “lay epidemiology” when parents ask for help from a health professional in making decisions about vaccination. It is important to consider “lay epidemiology” as an attempt of parents to make an extensive individual risk evaluation for their child. The health professional can be seen as an expert counselling the parents to evaluate and correct their “lay epidemiology” with help from scientific epidemiology.

#### Limitations

The focus group method for data collection is beneficial for exploring what people think of a health intervention and why [21]. Data are generated through interactions between the group members as they exchange attitudes and beliefs. Thus, the size of a focus group should be small enough for the members to be comfortable speaking but large enough to maintain group interactions. It can be argued that three or four member focus groups are too small; nevertheless, in this study, audio recordings and transcribed interviews demonstrated that group interactions occurred in the focus groups.

The small sample size of this study implies that the groups might not be representative of all expectant parents. Thus, the findings of this study should not be considered a complete list of arguments but, rather as examples of how some expectant parents make the decision of whether to vaccinate their child with BCG and their arguments for and against it. These findings might be useful for understanding parental decision making in regards to the BCG vaccine in general. Likewise the parents’ use of “lay epidemiology” in their risk evaluation might be relevant for the understanding of parental decision making regarding vaccines in general.

### Conclusion

Arguments in favour of receiving BCG vaccination are based on the possible beneficial effects of the vaccine on the immune system and that BCG is an old, well-established vaccine.

The findings of this study corroborate the results of studies in other western countries regarding parental concerns about undescribed negative side effects of vaccines and vaccine safety [2–4]. In addition, some parents were concerned about the scar caused by BCG immunization, whereas others evaluated the scar as a minor negative side effect compared to the possible beneficial effects of the vaccine.

The focus group research here suggests that “lay epidemiology” may form the basis for the parental decision of whether to allow their child to receive a BCG vaccination. Parents use their own interpretation of infections and atopic diseases that have occurred in their family to evaluate the risk of their child developing asthmatic bronchitis, eczema or other allergies or infectious diseases. This detailed knowledge about heritable factors should be seen as valuable additional knowledge by the health professional. Therefore, it would be ideal for parents if health professionals base their communication about the BCG vaccine on the parents’ individual risk evaluation.

It appears, based on the focus group discussions, that attitudes and beliefs about the causes and distribution of an illness within the parents’ families or their local network may influence the parents’ evaluation of the risk of their future child becoming sick. They also impact their assessment of the probability of negative side effects. Some of the concerns parents have about negative side effects could be considered unfounded.

A bad experience with negative side effects in a parent’s network bears more weight to that parent the closer the case is to the parent and the more similar it is to the situation of the parent. Therefore, when communicating with parents about vaccines, it may be beneficial for health professionals to ask parents about their concerns about and experiences with negative side effects.

Several parents emphasized that they need to talk with a health professional to make their decision, not because they need more information, but because they need help evaluating their individual risk based on “lay epidemiology”. It is important to recognize both the value element of “lay epidemiology” and the need for correction to the empirical element of “lay epidemiology” when health professionals counsel parents in making their decision about vaccines.

### Practice implications

“Lay epidemiology” promotes the understanding of how parents attempt to make an individual risk evaluation for their future child when deciding whether to allow their child to receive a BCG vaccine for the purpose of achieving non-specific effects on the immune system. In Denmark, the results may contribute to the understanding of suboptimal vaccination coverage rates of other vaccines as well. Accordingly, it is recommended that health professionals include this individual risk evaluation in their communication with parents not only about BCG, but other vaccines as well, and that they recognize both the empirical and the value element of “lay epidemiology”.

## Abbreviations

BCG: Baccile Calmette Guerin
